# User Experience, System Usability, and Feasibility of Two Novel Immersive Virtual Reality Memory Tasks for Cognitive Training: A Pilot Study

**DOI:** 10.3390/brainsci15121289

**Published:** 2025-11-29

**Authors:** Gaetano Tieri, Alberto Costa, Silvia Zabberoni, Erika Tenaglia, Maria Stefania De Simone

**Affiliations:** 1 Virtual Reality and Digital Neuroscience Lab, Department of Law and Digital Society, University of Rome UnitelmaSapienza, 00161 Rome, Italy; 2 Department of Economics, Psychology, Communication, Education, and Motor Sciences, Niccolò Cusano University, 00166 Rome, Italy; alberto.costa@unicusano.it (A.C.); s.zabberoni@hsantalucia.it (S.Z.); erika.ten09@gmail.com (E.T.); mariastefania.desimone@unicusano.it (M.S.D.S.); 3 Laboratory of Neuropsychology of Memory, Department of Clinical Neuroscience and Neurorehabilitation, IRCCS Santa Lucia Foundation, 00179 Rome, Italy

**Keywords:** immersive virtual reality, memory task, usability, feasibility, cognitive performance

## Abstract

**Background**: The implementation of effective, non-pharmacological interventions for enhancing cognitive function is a critical area of research. This pilot study evaluates the usability, feasibility, and acceptance of two novel immersive virtual reality (IVR) memory tasks designed for cognitive training. **Materials and Methods**: Thirty-three healthy young volunteers (mean age 20 ± 1.5 years) participated in a single session that included two IVR tasks: a “Virtual Face Name Memory Task” for long-term associative memory and a “Virtual Object Location Memory Task” for visuo-spatial working memory. The session, lasting approximately 30 min, was conducted using a Meta Quest 2 headset. To evaluate usability and feasibility, several standardized questionnaires were administered, including the User Satisfaction Evaluation Questionnaire, NASA Task Load Index, User Experience Questionnaire, Simulator Sickness Questionnaire, and System Usability Scale. Cognitive performance was measured through accuracy rates and the number of tasks completed. **Results**: Questionnaire results revealed an overwhelmingly positive user experience and high usability. Participants reported low frustration and a minimal incidence of cybersickness, confirming the procedure’s feasibility. Performance-wise, participants demonstrated high accuracy in immediate associative memory tasks (names: 80%, occupations: 95%) and visuospatial working memory tasks (change detection: 88–92%, localization: 90–95%). Associative memory performance declined after a 10 min delay (names: 49%, occupations: 59%) but improved significantly in the delayed recognition task (names: 76%, occupations: 88%). **Conclusions**: This pilot study provides compelling preliminary evidence for the usability and feasibility of two novel IVR memory tasks for cognitive training. The positive user experience, minimal cybersickness, and low frustration ratings indicate that the procedure is a feasible and engaging tool for cognitive intervention.

## 1. Introduction

Preventing disabilities associated with cognitive impairment has been declared a public health priority by the World Health Organization [1]. Cognitive decline, especially when affecting memory and executive functions [2], can interfere with motor function and dramatically compromise independence and safety during daily life activities [3,4]. It is therefore crucial to implement effective interventions focused on enhancing cognitive functions and functional independence, while also delaying cognitive decline and preserving existing abilities. However, recent decades have seen increasing healthcare costs and limited treatment duration and intensity, posing significant challenges to achieving optimal recovery. Furthermore, rehabilitation progress is often hindered by reduced patient engagement and shortages in both human and technological resources. These barriers underscore the urgent need to improve the quality and accessibility of cognitive and motor rehabilitation services [5].

Technological innovations offer novel avenues to engage individuals with cognitive impairment in simultaneous motor–cognitive training. Against this backdrop, Virtual Reality (VR) has emerged as a powerful and versatile tool in neurorehabilitation, providing immersive three-dimensional environments that closely mimic real-life scenarios. A key advantage of immersive VR (IVR) over conventional motor–cognitive training is its capacity to enhance participants’ motivation to engage in rehabilitation activities [6]. These controlled simulations not only elicit naturalistic patient responses but also enable therapists to safely replicate complex or dangerous situations.

Furthermore, IVR’s ability to foster realistic sensory–motor interactions between the user and the virtual environment significantly boosts engagement and ecological validity [7]. This is complemented by IVR-based experimental approaches that allow for the synchronized recording of behavioral, physiological (e.g., heart rate, skin conductance, respiratory rate, electromyography), and neural activity, due to their compatibility with a wide range of monitoring technologies [5].

A defining feature of IVR is its ability to generate a strong subjective feeling of physical presence within the virtual environment—a phenomenon known as the “Sense of Presence” [8]. This immersive state enables users to respond to virtual stimuli as though they were physically present, producing physiological reactions comparable to those elicited in real-world contexts [8,9]. Critically, the Sense of Presence engages neural circuits involved in sensorimotor integration and activates cortical networks that support sustained attention and cognitive processing [10].

Virtual reality has been successfully implemented in clinical interventions targeting a wide range of neurological conditions, including Alzheimer’s disease and Mild cognitive impairment [11,12], Parkinson’s disease [13,14,15], stroke [16] and unilateral spatial neglect [17].

In the context of the Alzheimer’s Disease (AD) continuum, analysis of recent literature suggests that VR-based interventions may offer benefits across cognitive function, physical function, and, to a lesser extent, quality of life in older adults with mild cognitive impairment (MCI) and AD dementia [18,19,20].

Despite this growing body of research supporting VR-based treatments in the prodromal and full-blown disease stages of AD, its application among individuals in the preclinical stage—such as those with Subjective Cognitive Decline (SCD)—remains notably limited. SCD is clinically characterized by a self-perceived worsening of cognition in the absence of objective cognitive impairment on standardized neuropsychological testing or in activities of daily living [21]. It is widely associated with an increased risk of progression to MCI and subsequent dementia [22]. Moreover, these individuals often present with subclinical symptoms, such as anxiety and depression, which further contribute to their vulnerability [23].

To date, evidence regarding the efficacy of VR-based cognitive training in individuals with SCD remains scarce. To the best of our knowledge, only two previous studies have investigated the efficacy of this intervention in this population, yielding promising results [24,25]. In particular, Kang et al. [25] evaluated multidomain cognitive training in a VR environment, showing significant improvements across a wide range of domains, including attention, executive function, processing speed, and working memory. Similarly, Arlati et al. [24] investigated the acceptance and usability of an immersive VR shopping task, demonstrating that individuals with SCD enjoyed the VR experience and reported low levels of cybersickness.

However, evidence is still limited and some methodological shortcomings (e.g., the absence of a randomized controlled trial design and training that does not specifically target core SCD cognitive deficits) need to be addressed. This highlights a significant research gap that warrants further exploration (see Maggio et al. [26] for a recent systematic review).

In this regard, we have recently developed a randomized controlled trial aimed at evaluating the effectiveness of a novel, multi-component intervention—combining cognitive training with health education—delivered through immersive VR and telemedicine [27]. This accessible and cost-efficient approach holds considerable potential for early cognitive intervention and primary prevention in populations at elevated risk for dementia.

Crucially, when designing and developing IVR training concepts, it is paramount to consider the intended users’ characteristics, needs, experiences, and perspectives. This user-centered approach is vital for ensuring the quality and practical utility of the final training concept [28,29].

In light of the above, this pilot study aimed to evaluate the feasibility, system usability, and acceptance of the two novel Immersive Virtual Reality (IVR) memory tasks specifically designed and developed for cognitive training of individuals with SCD (for more details, see [27]) in order to highlight potential issues that needed to be improved before conducting the planned RCT on the target clinical population. For this purpose, a sample of healthy young subjects underwent a single session of the cognitive training described in [27], which included the two virtual memory tasks.

The first, the Virtual Face-Name Memory Task, is a long-term associative memory task assessing the ability to learn and recall name-face-occupation associations, both immediately and after a 10 min delay. The second, the Virtual Object-Location Memory Test, is a visuospatial working memory binding test evaluating the ability to integrate and retain multiple features within an object and in relation to its spatial context. Empirical findings demonstrate that individuals with SCD exhibit objective deficits in these specific and more demanding cognitive processes when compared with healthy individuals without SCD [30]. Our primary objective was to evaluate the user-centered aspects of these proposed IVR-based cognitive tasks, providing multifaceted insights into their design efficacy and cognitive demands. To achieve this, we employed a suite of standardized, self-report questionnaires administered immediately after the IVR task session. These assessed participant experience, usability, perceived mental workload, and potential adverse VR-induced side effects. As a secondary aim, we evaluated participant performance on the IVR tasks. Our focus was to analyze the data collected on how participants interacted with and performed within the complex virtual environment, with a particular emphasis on key cognitive functions like recall and recognition memory, change detection, and spatial location memory. To this end, we quantified performance using metrics such as accuracy rates and the total number of tasks completed. This analysis will provide preliminary insights crucial for adapting the virtual experience for cognitive training in individuals with SCD.

## 2. Materials and Methods

### 2.1. Participants

Thirty-three undergraduate psychology students at the Niccolò Cusano University of Rome (Italy) participated in the study (8 males and 25 females). We recruited them on a voluntary basis through an advertisement in class. Given the voluntary and exploratory nature of this pilot study on usability, stringent formal exclusion criteria were not applied. Thirty-two percent of participants stated they had never used virtual reality technologies before (*n* = 11), and 55 percent of participants stated they had minimal experience with immersive virtual reality (*n* = 18). Only 12% of the sample stated they had greater experience with IVR technologies (*n* = 4), all of whom had used head-mounted displays and primarily for playing video games. The experimental protocol received approval from the Territorial Ethics Committee of Lazio Area 5, Italy (experiment register N.81/SL/23) and was conducted under the ethical standards of the 2013 Declaration of Helsinki. All participants provided their informed consent prior to taking part in the study.

### 2.2. Hardware and Software Implementation

Experimental tasks were developed in Unity 2022.3.5f1 and subsequently deployed as a standalone Android Package Kit (APK) compatible with the Meta Quest 2 head-mounted display (HMD). The HMD features a resolution of 1.832 × 1.920 pixels per eye, a 72 Hz refresh rate, and 6 degrees of freedom (DoF). A user-friendly interaction system was developed using custom Unity C# scripts, incorporating ray-cast object selection and trigger-based interactions with the user interface (UI). Additional custom C# scripts managed task stimulus presentation, timing control, and data recording. Data were collected directly within the HMD, with specific .txt files saved for each participant, containing their unique ID number and experimental data.

### 2.3. Virtual Environments

Virtual stimuli, objects, and environments were designed using 3DS Max 2022 (Autodesk, Inc., San Rafael, CA, USA) for modeling and Unity 2022.3.5f1 for environment development and implementation. Two distinct virtual environments were created, i.e., the Virtual Face Name Memory task and the Virtual Object Location Memory task.

The Virtual Face Name Memory task comprised a minimalist virtual environment: a darkened spatial volume featuring a single, centrally located cylindrical column as its primary visual focal point. This column was illuminated by a focused spotlight. Virtual faces were presented serially at the apex of the column, while corresponding interactive user interface (UI) panels (approximately 60 × 30 cm), containing various instructions and questions, appeared directly in front of the column to facilitate participant responses. The 3D facial models were derived from the Microsoft Rocketbox avatar library, from which forty avatars (20 male) were specifically selected. These models were optimized in 3DS Max 2022 by removing their bodies and bones, then imported into Unity as .obj files with a 1:1 scale. For each virtual face, a specific name and occupation were randomly assigned. These assignments utilized a pool of 40 common Italian names (20 male) and 20 gender-matched occupations. A customized C# script, developed within Unity, was used for presenting this associated information on the UI.

Regarding the Virtual Object Location Memory task, the virtual environment consisted of a large living room, approximately 8 × 8 m in size and modeled at a 1:1 scale. This room featured a centrally located table and several empty shelving units placed on the front wall. Within these shelves, a variable number of 3D objects were presented, adjusted according to task difficulty. These 3D objects were designed and optimized using Autodesk 3DS Max 2022 and Unity. Specifically, we created 30 3D objects across 10 semantic categories, with three objects per category. These categories included: Plants, Lamps, Vases, Balls, Stuffed Animals, Large Chess Models, Framed Photos, Bottled Drinks, Books, and Fruit Baskets. Above the centrally located 3D table, and directly in front of the participants’ viewpoint, the user interface (UI) displayed activity-specific instructions and questions (see subsequent paragraph for details).

### 2.4. Experimental Procedure

**Figure 1:**
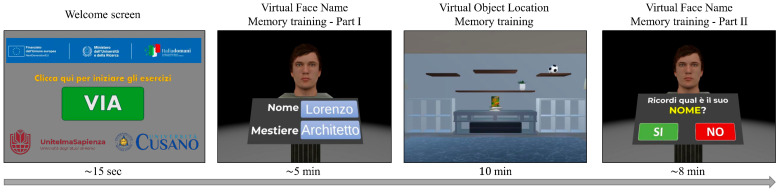
**Temporal organization of the experimental procedure**. The figure depicts the sequential flow of the experiment, including the Welcome screen (Left panel—translated from Italian: *“Click here to start the exercises*”), Virtual Face Name memory task Part 1 (Left-central panel—translated from Italian: *“Name: Lorenzo*”; “*Occupation: Architect*”), Virtual Object Location memory task (Right-central panel), and Virtual Face Name memory task Part 2 (Right panel—translated from Italian: “*Do you remember his name?*”).

Each participant was assessed individually in a single session lasting approximately 30 min. During this session, participants were comfortably seated in a quiet room and equipped with an HMD, allowing for immersion in the virtual environments from a first-person viewpoint. The procedure consisted of two virtual memory tasks (i.e., the Virtual Face Name memory task and the Virtual Object Location memory task), articulated in different sequential phases, as illustrated in Figure 1.

**Figure 2:**
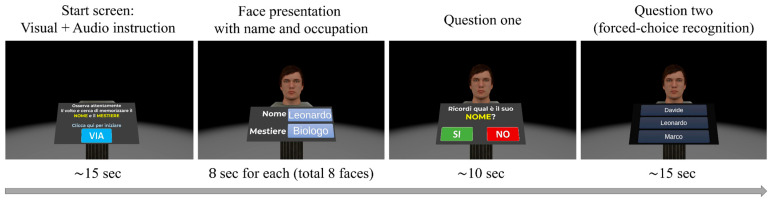
**Temporal organization of the Virtual Face Name memory task Part 1**. Left panel: The start screen displaying visual and audio instructions. Translated from Italian: “*Look carefully at the face and try to memorize the name and the occupation. Click here to get started*”. Left-central panel: Example of a 3D face presented with an associated name and occupation (e.g., Name: Leonardo; Occupation: Biologist). Each face was displayed individually for 8 s, and this sequence was presented twice. Right-central panel: The initial question posed to participants (translated from Italian: “*Do you remember his*/*her name*?”). Right panel: A three-alternative forced-choice recognition task for the name. The initial question and recognition tasks (right-central and right panels) were repeated for each face, first for names and then for occupations.

Specifically, the experiment began with a Welcome screen (Figure 1, first panel) that served two purposes. First, it familiarized participants with ray-cast interaction, controlled using their dominant-hand Meta Quest controller. Second, it enabled spatial calibration, ensuring the UI panel was centered directly in front of them. After clicking the “Start” UI button (labeled “Via” in Italian), the Virtual Face Name memory task Part 1 was administered (Figure 1, second panel). This task assessed the ability to learn and immediately retain a series of face-names-occupation associations. In particular, participants were presented with 8 virtual faces, balanced by sex (female/male) and age (young adults/seniors). Each face was paired with a unique name and occupation and displayed individually for 8 s (Figure 2, left central panel). Participants were instructed to memorize these face-name-occupation associations. Immediately following this initial exposure, a second learning phase was administered, during which the same set of face-name-occupation pairs was presented individually for 8 s each, but in a different randomized order. After this second learning phase, the previously learned faces were again presented individually. For each face, participants were first asked, “*Do you remember his*/*her name*?” (Possible response: yes/no). Subsequently, they performed a three-alternative forced-choice recognition task, selecting the correct name (target) from two incorrect options: a lure (a name previously paired with a different face) and a distractor (a novel, unstudied name) (Figure 2). The same procedure was then used to assess immediate memory for occupations associated with each face (question: “*Do you remember his*/*her occupation*?” followed by the three-alternative forced-choice recognition task).

**Figure 3:**
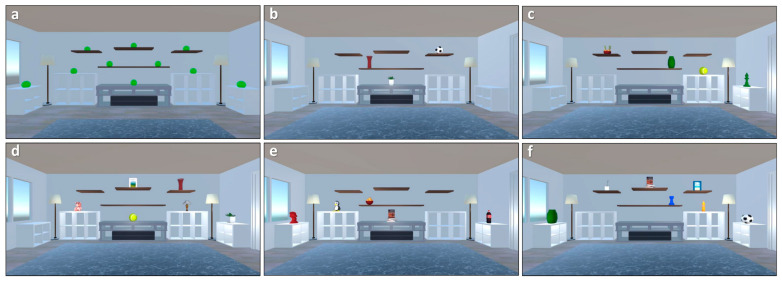
**Increased Task Difficulty and Object Placement**. This figure illustrates the increasing task difficulty across trials in the Virtual Object Location memory task. Panels (**b**–**f**) depict trials with a progressively growing number of objects (from 3 to 7) that participants were required to memorize. Panel (**a**) highlights the symmetrical spatial layout of all potential object placement locations within the environment. Note that the green spheres in Panel (**a**) are purely illustrative to denote positions and were not present during experimental trials.

Virtual Face Name Part 1 concluded with a black screen covering the entire virtual environment. Subsequently, participants were immersed in the next memory task: the Virtual Object Location memory task (Figure 1, third panel). In the study phase, participants were instructed to carefully observe a virtual scenario (consisting of a room containing an increasing number of everyday objects in different spatial locations) and memorize the objects along with their spatial locations. The number of objects shown in the room progressively increases (from 3 to 11) as the difficulty level of the task increases in accordance with participants’ performance. Figure 3 illustrates examples of the virtual scenario with different task difficulty, showing trials with an increasing number of objects displayed.

**Figure 4:**
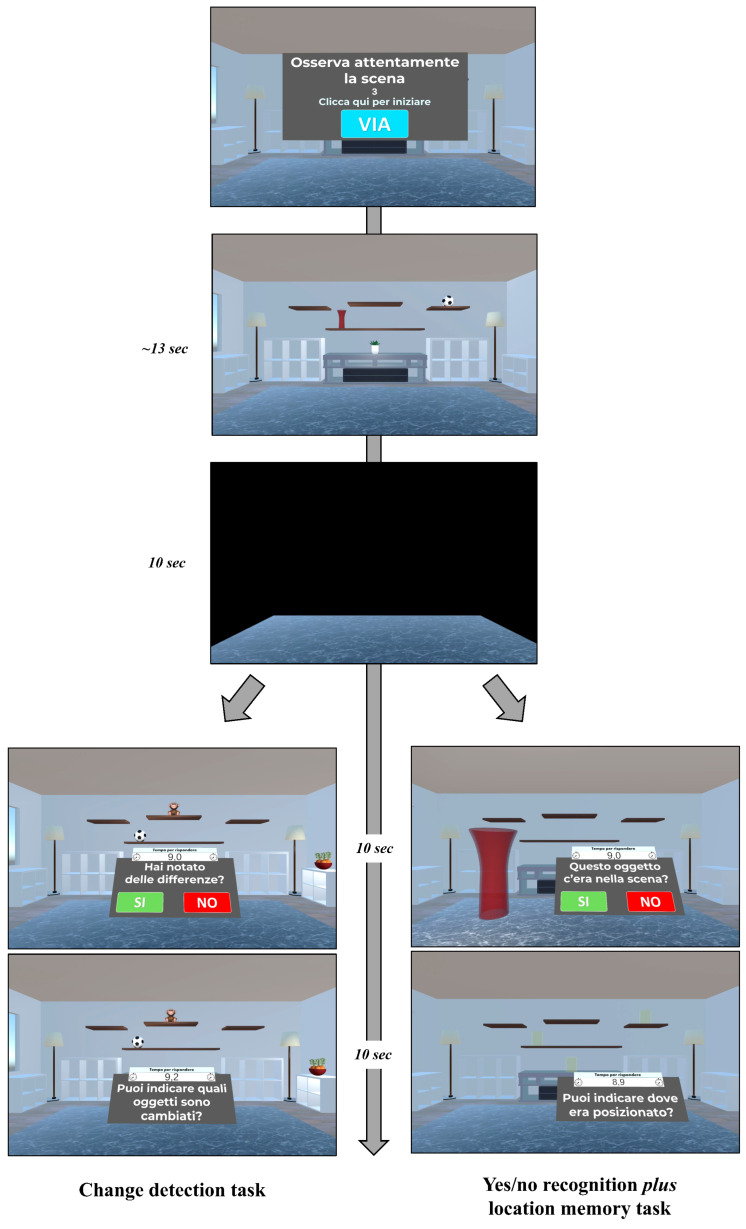
Temporal Organization of the Virtual Object Location Memory Task, encompassing a Study phase and a Test phase. Study phase. (Upper Panel): Participants received visual and auditory instructions (translated from Italian: “*Carefully observe the scene. Click here to get started*”) to begin the task. This was followed by the observation of a virtual scenario for 13 s, during which participants were instructed to memorize the objects and their precise locations (Middle Panel). A 10 s black screen separated the Study and Test phases (Third Panel). Test phase. Change Detection Format (Lower Left Panels): The room reappeared and participants answered two sequential questions: first, “*Did you note any differences*?” (Yes/No response), and subsequently, “*Can you indicate which objects have changed*?”. Yes/No Recognition Plus Location Memory Format (Lower Right Panels): A single object (e.g., the red jar shown) was presented centrally. Participants first responded to the question, “*Was this object present in the study phase*?” (Yes/No response). If the object was correctly recognized as a target, they then indicated its original position, “*Can you indicate where the object was located*?”.

Following a 13 s exposure, the virtual room disappeared. After a 10 s blank screen serving as a retention interval, the test phase was administered. This comprised two test formats of increasing difficulty: a change-detection task (Levels 1–3) and a yes/no recognition plus location memory task (Levels 4–12), as illustrated in Figure 4.

In particular, the change-detection task involved the virtual room reappearing under one of three conditions: (i) no-change (all objects remained in their original positions), (ii) position-change (the same objects were present but in different locations) and (iii) object-change (different objects occupied the original positions). Participants were required to indicate whether the objects and their locations were the same or different between the study and test phases by responding “Yes” or “No.” If a change was correctly identified, participants were asked to click on the altered objects or locations to specify the change (Figure 4, lower left panels).

When participants achieved two consecutive correct responses, the number of displayed objects increased. All participants started at the easiest Level 1 (3 objects). After two consecutive successful trials (no errors), they advanced to the next level (Level 2: 4 objects). Upon successfully completing the 5-object level (Level 3), the task was adapted to the yes/no recognition and location memory test format.

In the yes/no recognition plus location memory task, after the black screen, the room reappeared and a series of virtual objects were presented individually in front of the participants. This series included items from the study phase (targets), new items not previously seen (foils), and new items perceptually similar to those seen during the study phase, but not identical (lures). For each stimulus, participants first performed a yes/no recognition judgment, indicating whether the displayed item was a studied object or an unstudied one. If an object was correctly recognized as a target, participants were then prompted to indicate its original location (localization memory) (Figure 4, lower right panels—“*Can you indicate where it was positioned*?”). Similar to the prior task, difficulty was dynamically calibrated according to performance. All participants started with 3 objects (Level 4). Upon achieving two consecutive correct trials (no errors), the number of displayed objects increased by one, up to a maximum of 11 objects displayed (Level 12).

For the Virtual Object Location memory task, participants were asked to perform as many trials as possible in 10 min. After this time, the task concluded with a black screen covering the entire virtual environment.

**Figure 5:**
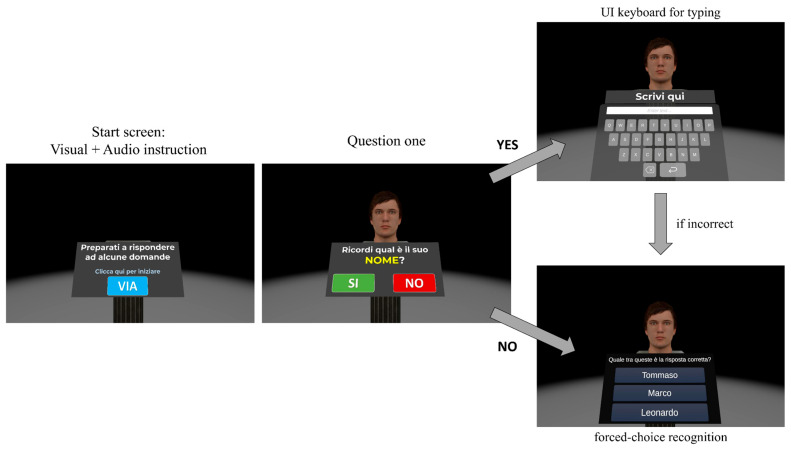
**Temporal organization of the Virtual Face Name memory task, Part 2**. Left panel: The start screen displays visual and audio instructions for the task. Translated from Italian: “*Be prepared to answer some questions. Click here to get started*”. Central panel: The initial question presented to participants (i.e., “*Do you remember his*/*her name*/*occupation*?”). Upper right panel: If the answer was “YES,” a UI keyboard appeared for typing the name or occupation. Lower right panel: If the cued recall was incorrect or the answer was “NO,” a three-alternative forced-choice recognition task was presented (translated from Italian: “*Which of these is the correct answer? Alternatives: Tommaso, Marco, Leonardo*”.

Subsequently, participants were immersed in the last task, the Virtual Face Name memory task Part 2 (Figure 1, fourth panel). This task assessed the delayed cued-recall and recognition abilities of the face-name-occupation associations studied in Task Part 1. Specifically, previously studied faces were presented individually. As illustrated in Figure 5, for each face, participants were first asked, “*Do you remember his*/*her name*?” If the answer was “yes,” a UI keyboard appeared for typing the name (delayed cued recall). If the answer was “no” or the entered name was incorrect, a three-alternative forced-choice recognition UI then appeared. In this task, participants were asked to select the correct name (target) from a novel name (distractor) and a lure (a name paired with a different face) (Figure 5, right panel). The same procedure was used to assess delayed cued recall and recognition of occupations. The experiment concluded once participants answered all questions regarding face-name-occupation associations.

### 2.5. Administration Procedure

The task administration order was fixed for each subject. This methodological choice was essential for the appropriate assessment of long-term associative memory (Virtual Face-Name Memory), which required a fixed temporal separation between the immediate and delayed phases. Specifically, the 10 min working memory task (Virtual Object Location Memory task) was deliberately and uniformly administered as an interference period between the immediate assessment (Part 1) and the delayed assessment (Part 2) of the Face-Name task, ensuring the validity of the delayed recall measure.

### 2.6. Evaluation of Performance on Experimental Procedure

Participant performance on the IVR experimental procedure was recorded. Specifically, for the Virtual Face Name memory task Part 1, collected measures included: (i) the number of names correctly recognized in the three-alternative forced-choice recognition task (range: 0–8), and (ii) the number of occupations correctly recognized in the three-alternative forced-choice recognition task (range: 0–8). For the Virtual Face Name memory task Part 2, collected measures included: (i) the number of names correctly retrieved in the delayed cued recall, as correctly typed using the UI keyboard (range: 0–8); (ii) the number of occupations correctly retrieved in the delayed cued recall, as correctly typed using the UI keyboard (range: 0–8); (iii) the number of names correctly recognized in the three-alternative forced-choice recognition task; and (iv) the number of occupations correctly recognized in the three-alternative forced-choice recognition task. Since in Task Part 2 the three-alternative forced-choice recognition was administered only for the faces whose name and/or occupation was not retrieved in delayed cued-recall, we calculated recognition accuracy for names and occupations, respectively, by applying the following formula for each subject: n of correct recognitions/(8—delayed cued recall score). The resulting indexes of recognition for names and occupations ranged from 1 (i.e., correct recognition of all names/occupations not retrieved in the delayed cued-recall trial) to 0 (i.e., no advantage in performing the recognition with respect to the delayed cued recall).

Regarding the Virtual Object Location memory task, performance data for the change-detection memory format included: (i) accuracy in change detection, defined as the number of correct responses to the question “*Did you note any differences*?”, which encompasses “yes” responses when a change occurred and “no” responses when no change occurred; and (ii) the number of object or location changes correctly identified in response to the question “*Can you indicate which objects have changed*?”

For the yes/no recognition plus location memory task, performance data comprised: (i) accuracy in object recognition, defined as the number of correct responses to the question “*Was this object present in the scene*?”, encompassing “yes” responses for target objects and “no” responses for distractor objects (lure or foils); and (ii) accuracy in object location, defined as the number of correct responses to the question “*Can you indicate where it was positioned*?”.

### 2.7. Post-Experimental Questionnaires

Immediately following the completion of the IVR session, participants were invited to complete five standardized self-report questionnaires to evaluate the usability and feasibility of the proposed procedure: the User Satisfaction Evaluation Questionnaire (USEQ [31]), NASA Task Load Index (NASA-TLX [32]), User Experience Questionnaire (UEQ [33]), Simulator Sickness Questionnaire (SSQ [34]), and System Usability Scale (SUS [35,36]). Questionnaire administration lasted approximately 15 min for each participant and took place outside of the VR environment.

The USEQ and NASA-TLX questionnaires were employed to assess participants’ self-perception of usability and the perceived workload demands associated with the virtual reality tool, respectively. The USEQ [31] comprises six questions (e.g., “*Did you enjoy your experience with the system*?”, “*Was the system easy to use*?”), each rated on a five-point Likert scale ranging from 1 to 5. Each question evaluates a specific dimension, including self-perceived satisfaction (“*Experience enjoyment*”), efficacy (“*Successful use*”), efficiency (“*Ability to control*”), ease of use (“*Clarity of information*”), fatigue (“*Discomfort*”), and the utility of the performed exercise (“*Perceived utility*”).

The NASA-TLX [32] consists of six questions, each assessed using a ten-point numerical rating scale (e.g., “*How physically demanding was the task*?”, “*How hard did you have to work to accomplish your level of performance*?”). Each question evaluates a specific dimension, including Mental demand, Physical demand, Temporal demand, Effort, Performance, and Frustration/Stress level.

The UEQ [33] was employed to assess the acceptability of the virtual reality tool. This questionnaire comprises 26 items distributed across six scales: *Attractiveness*, *Efficiency*, *Perspicuity*, *Dependability*, *Stimulation*, and *Novelty*. Responses for each item were recorded on a 7-point Likert-type scale, ranging from −3 (very negative) to +3 (very positive), representing a gradient between contrasting attributes (e.g., “*complicated—easy*”, “*inefficient—efficient*”, “*boring—exciting*”). Participants rated the experimental procedure by selecting the appropriate point on this scale. Values above 0.8 are considered indicative of a positive evaluation.

The SSQ [34] was employed to assess possible negative side effects of VR use (i.e., cybersickness). This validated 16-item self-report questionnaire assesses the severity of individual symptoms, including, but not limited to, general discomfort, fatigue, headache, nausea, and vertigo. Items are rated on a 4-point scale, ranging from 1 (Absent) to 4 (Strong), to indicate symptom severity.

The SUS [35,36] was employed to assess the subjective evaluation of the interface technology’s usability. This validated 10-item instrument quantifies both learnability and user satisfaction (e.g., “*I think that I would like to use this system frequently*”, “*I found the various functions in this system were well integrated*”). Items are rated on a 5-point Likert scale, ranging from 1 (Strongly Disagree) to 5 (Strongly Agree), and address aspects such as ease of use, technical consistency, and suitability for continued use.

## 3. Results

### 3.1. Usability and Feasibility of the IVR Procedure

**Figure 6:**
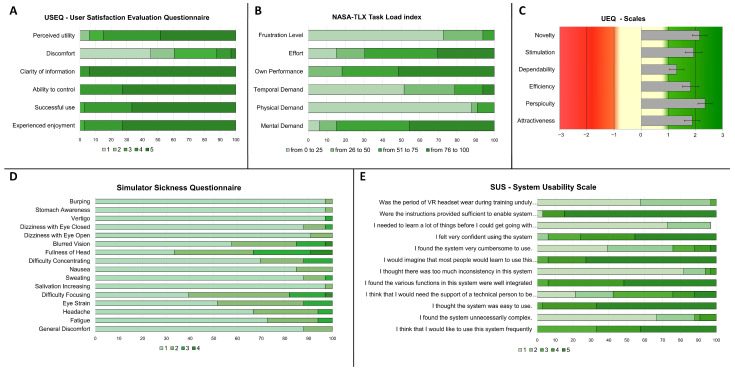
**Participant feedback on the immersive virtual reality (IVR) memory tasks**. This figure displays the aggregated results from various self-report questionnaires administered to participants after the IVR session. Data are presented as mean percentages (**A**,**B**,**D**,**E**) or mean scores (**C**).

The USEQ results indicated a high overall satisfaction (Figure 6A). Specifically, for the “*Experience enjoyment*” dimension (mean ± SE: 4.70 ± 0.09), 72.7% of participants provided the maximum rating of 5, while 24.2% rated it 4 and 3% rated it 3. Similar trends were observed across other dimensions: “*Successful use*”(mean ± SE: 4.64 ± 0.10) had 66.7% of participants rating 5 and 30.3% rating 4 and 3% rated 3; “*Ability to control*” (mean ± SE: 4.73 ± 0.08) had 72.7% rating 5 and 27.3% rating 4; and “*Clarity of information*” (mean ± SE: 4.94 ± 0.04) received the highest ratings with 93.9% at 5 and 6.1% at 4. “*Perceived utility*” (mean ± SE: 4.27 ± 0.15) showed 48.5% of participants rating 5, 36.4% rating 4, 9.1% rating 3, and 6.1% rating 2. Regarding the dimension “*Discomfort*” (mean ± SE: 2.09 ± 0.21) 45.5% of participants rated 1 (no discomfort), 15.2% rated 2, 27.3% rated 3, 9.1% rated 4, and 3% rated 5.

The NASA-TLX results indicated a good level of perceived workload demands (Figure 6B). Specifically, for the “*Mental Demand*” (mean ± SE: 71.76 ± 3.45) dimension, 45.5% of participants provided ratings between 76 and 100, while 39.4% rated between 51 and 75. Smaller proportions rated between 26 and 50 (9.1%) and between 0 and 25 (6.1%) (Note that higher values on this dimension indicate a greater mental effort required for task execution). Similar results have been observed for other dimensions. For the “*Performance*” dimension (mean ± SE: 74.55 ± 3.23), 51.5% of participants provided ratings between 76 and 100, 30.3% rated between 51 and 75, while smaller proportions rated between 26 and 50 (18.2%). For the “*Effort*” dimension (mean ± SE: 60.58 ± 4.48), 30.3% of participants provided ratings between 76 and 100, 39.4% rated between 51 and 75, 15.2% rated between 26 and 50 and 15.2% rated between 0 and 25. Opposite trend was observed for other dimension “*Physical Demand*” (mean ± SE: 16.15 ± 2.72) (87.9% rated between 0 and 25, 3% rated between 26 and 50 and 9.1% rated between 51 and 75) and “*Temporal Demand*” (mean ± SE: 32.52 ± 4.26) (51.2% rated between 0 and 25, 27.3% rated between 26 and 50, 15.2% rated between 51 and 75 and 6.1% rated between 76 and 100). For the “*Frustration*/*Stress Level*” dimension (mean ± SE: 16.12 ± 3.22), 72.7% of participants provided ratings between 0 and 25 (lower frustration level), 21.2% rated between 26 and 50 and 6.1% rated between 51 and 75. The overall mental workload score was calculated by means of the unweighted combination method, as reported by [37]. The resulting total score was 37 (on a range of 0 to 100), which indicates a moderate level of perceived mental workload associated with the task performance.

Results on the UEQ indicated an overall positive evaluation across all dimensions of the experience. Specifically, the mean values for each dimension were: *Attractiveness* 1.88, *Efficiency* 1.82, *Perspicuity* 2.36, *Dependability* 1.31, *Stimulation* 1.94, and *Novelty* 2.16. All scores ranged from −3 (very negative) to +3 (extremely positive), with values above 0.8 considered a positive evaluation [33] (mean and variance are reported in Supplemental Materials Table S3).

The Simulator Sickness Questionnaire (SSQ) results indicated a very low incidence of virtual reality (IVR) induced side effects, as illustrated in Figure 6D. Almost all participants rated each symptom with scores of 1 (Absent) or 2 (Slight), while only a small percentage reported symptoms with a severity of 3 (Moderate) or 4 (Strong) (mean, standard deviation and standard error are reported in Supplemental Materials Table S5). Mean value of SSQ subscales, including Nausea, Oculomotor, and Disorientation and the total score were obtained by following the Computation of SSQ Scores reported in [34,35,36,37,38]. Results confirm the low incidence of IVR induced side effects in all subscale, i.e., Nausea (mean ± SE: 8.96 ± 1.85), Oculomotor (mean ± SE: 25.04 ± 1.85) Disorientation (mean ± SE: 41.34 ± 5.43) and, subsequently, in Total Score (mean ± SE: 28.97 ± 3.22) (Supplemental Materials Table S6).

The SUS results indicated a positive overall evaluation of the interface technology’s usability (Figure 6E, Supplemental Materials Table S4). Items reflecting high perceived usability included: “*I thought the system was easy to use*” (66.7% rated 5, 30.3% rated 4, 3% rated 3); “*I would imagine that most people would learn to use this system very quickly*” (72.7% rated 5, 21.2% rated 4, 6.1% rated 3); “*I felt very confident using the system*” (45.5% rated 5, 30.3% rated 4, 18.2% rated 3, 6.1% rated 2); and “*I found the various functions in this system were well integrated*” (51.5% rated 5, 42.4% rated 4, 6.1% rated 3). Additionally, strong agreement was observed for the item “*I think that I would like to use this system frequently*” (42.4% rated 5, 24.4% rated 4, 33.3% rated 3). Conversely, items indicating low perceived usability were rated accordingly: “*I found the system unnecessarily complex*” (66.7% rated 1, 21.2% rated 2, 3% rated 3, 9.1% rated 4); “*I thought there was too much inconsistency in this system*” (81.8% rated 1, 12.1% rated 2, 3% rated 3, 3% rated 4); “*I found the system very cumbersome to use*” (39.4% rated 1, 36.4% rated 2, 12.1% rated 3, 9.1% rated 4, 3% rated 5); and “*I needed to learn a lot of things before I could get going with this system*” (72.7% rated 1, 24.2% rated 2). The item “*I think that I would need the support of a technical person to be able to use this system*” received mixed responses (21.2% rated 1, 21.2% rated 2, 33.3% rated 3, 12.1% rated 4, 12.1% rated 5). As an extra item, “*Were the instructions provided sufficient to enable system utilization*?” received overwhelmingly positive feedback, with 84.4% of participants rating 5, 12.1% rating 4, and 3% rating. The SUS score was calculated according to the methods reported in [35]. The result yielded a total score of 77 (on a range of 0 to 100), which confirms a good perceived usability of the system.

### 3.2. Participants’ Performance on IVR Tasks

#### 3.2.1. Virtual Face Name Memory Task

**Figure 7:**
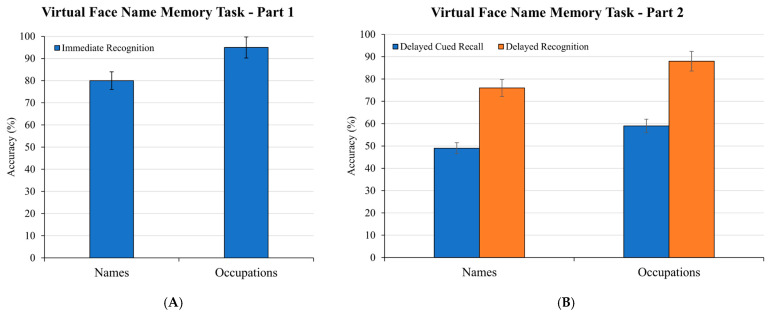
**Performance on the Virtual Face Name Memory Task**. (**A**) displays accuracy for names and occupations in the immediate recognition (Part 1). (**B**) presents performance for names and occupations in the delayed cued recall and delayed recognition (Part 2) after a 10 min interval. Error bars represent the standard error of the mean.

Performance on the Virtual Face Name Memory Task was assessed separately for task Part 1 (immediate recognition) and Part 2 (delayed cued recall and recognition), as illustrated in Figure 7.

Regarding Part 1, the average percentage of correct responses provided on the immediate three-alternative forced-choice task was considered as an accuracy measure for names and occupations, respectively. As shown in Figure 7A, participants demonstrated high accuracy in the immediate three-alternative forced-choice recognition task. In fact, they correctly recognized target names associated with studied faces with an average accuracy of 80%. Performance for recognizing occupations was even higher, reaching an average of 95% correct responses.

Regarding Part 2, Figure 7B illustrates participants’ delayed memory performance for face-name and face-occupation associations following a 10 min interval. As shown, two accuracy scores were considered for names and occupations, respectively: delayed cued recall (percentage of names or occupations correctly retrieved via UI keyboard input) and delayed recognition (percentage of names or occupations correctly recognized in the subsequent three-alternative forced-choice task). In the delayed cued recall, participants achieved 49% accuracy in recalling the correct name associated with each face, and 59% accuracy for occupations. Memory performance notably improved in the delayed recognition task. In fact, in the three-alternative forced-choice format, participants demonstrated 76% accuracy in recognizing target names and 88% accuracy in recognizing occupations associated with the previously studied faces.

#### 3.2.2. Virtual Object Location Memory Task

**Figure 8:**
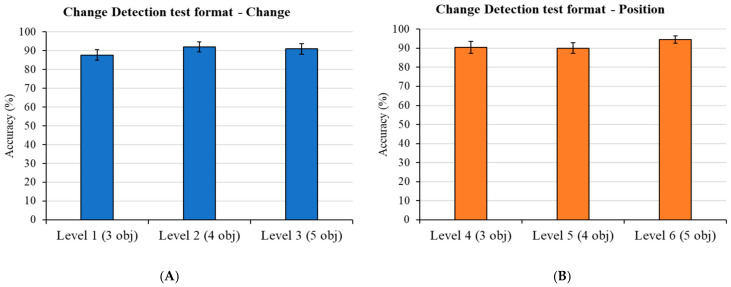
**Mean Accuracy in the Virtual Object Location Memory Task: Change-Detection Test Format**. (**A**) displays the mean percentage of correct responses to the initial question, “*Did you note any differences*?”, across three difficulty levels: Level 1 (3 objects), Level 2 (4 objects), and Level 3 (5 objects). (**B**) illustrates the mean accuracy for identifying the positions of changed objects in response to the question, “*Can you indicate which objects have changed*?”, for the same difficulty levels. Error bars represent standard errors of the mean.

Mean performance scores on the change-detection test format (Levels 1 to 3) of the Virtual Object Location memory task are illustrated in Figure 8. Participants demonstrated high accuracy in recognizing changes across all three difficulty levels. In fact, for the initial question, “*Did you note any differences*?”, the mean percentage of correct responses was 88% in Level 1 (3 objects), 92% in Level 2 (4 objects), and 91% in Level 3 (5 objects) (Figure 8A). Similarly, high performance was observed for the subsequent question, “Can you indicate which objects have changed?” Participants correctly identified the positions of the changed objects with 90% accuracy for Level 1, 90% for Level 2, and 95% for Level 3 (Figure 8B).

**Figure 9:**
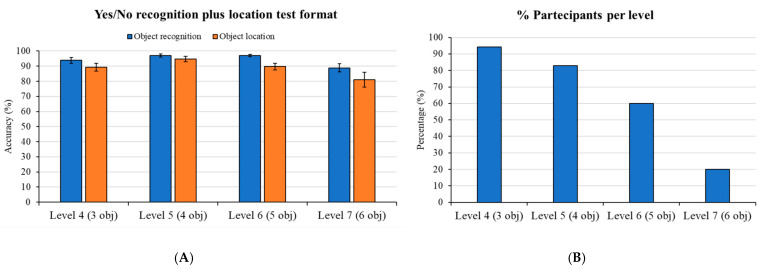
(**A**): Mean Accuracy in the Virtual Object Location Memory Task: Yes/No Recognition Plus Location Memory Format. This graph illustrates participants’ mean performance scores on the yes/no recognition plus location memory test format across four difficulty levels. The blue bars represent the mean accuracy for object presence recognition (i.e., correct responses to “Was this object present in the scene?”), while the orange bars display the mean accuracy for object location recall (i.e., correct responses to “Can you indicate where it was positioned?”). Error bars represent standard errors of the mean. (**B**): Percentage of Participants Successfully Completing Each Difficulty Level of the Virtual Object Location Memory Task. This graph illustrates the progressive decrease in the percentage of participants who successfully advanced to and completed each higher difficulty level within the time-limited (10 min) task.

Figure 9A illustrates performance on the yes/no recognition plus location memory test format (Levels 4 to 12). As shown in Panel A, participants demonstrated high accuracy in object recognition across all difficulty levels. In fact, for the question “*Was this object present in the scene*?”, the mean percentage of correct responses was 94% for Level 4 (3 objects), 97% for Level 5 (4 objects), 97% for Level 6 (5 objects), and 89% for Level 7 (6 objects). Similarly, high performance was observed for object location recall, as depicted in Panel B. In fact, for the question “*Can you indicate where it was positioned*?”, the mean percentage of correct responses was 89% for Level 4, 95% for Level 5, 90% for Level 6, and 81% for Level 7.

However, in this time-limited task (where participants aimed to complete as many levels as possible in 10 min), the percentage of participants who successfully advanced to and completed each level decreased progressively as difficulty increased. As illustrated in Figure 9B, in fact, 94% of participants successfully completed Level 4, 83% completed Level 5, 60% completed Level 6, and only 20% completed Level 7.

## 4. Discussion

This pilot study aimed to evaluate the user-centered aspects and feasibility of two novel immersive VR tasks designed for cognitive training [27]. To achieve this, we administered a comprehensive set of standardized self-report questionnaires to a sample of healthy young adults. These measures were used to assess the overall participant experience, system usability, perceived mental workload, and potential adverse VR-induced side effects.

As a secondary aim, we evaluated participants’ performance on the IVR tasks. We specifically assessed how they interacted with and performed in the complex virtual environment, focusing on key cognitive processes such as associative recall and recognition memory, change detection, and spatial location memory.

### 4.1. Usability and Feasibility of the IVR Procedure

The findings from the User Experience Questionnaire (USEQ), User Experience Questionnaire (UEQ) and the System Usability Scale (SUS) consistently revealed an overwhelmingly positive participant experience and high usability of the IVR procedure.

In particular, the USEQ data indicated high overall satisfaction, with participants reporting highly positive ratings across all dimensions. Specifically, the “Experience enjoyment” ratings highlighted that the IVR task design positively affected the self-perceived satisfaction, with a large percentage of participants reporting maximum or near-maximum enjoyment. Similarly, the high ratings for “Successful use” and “Ability to control” suggest that the IVR tasks were perceived as highly efficacious and efficient. The “Clarity of information” dimension also received high ratings, underscoring the easy-to-use and intuitive nature of the IVR tasks. Furthermore, “Discomfort” ratings suggested an overall absence of fatigue, with a substantial percentage of participants reporting very low or no discomfort. While “Perceived utility” remained largely positive, only a small percentage of participants rated its utility as less pronounced.

The UEQ results further corroborated the findings on usability, with positive evaluations across all dimensions, indicating strong overall user appreciation. Specifically, high ratings for “Attractiveness” suggested that participants found the IVR tasks to be both appealing and enjoyable. The scores for “Perspicuity”, which gauges ease of understanding and learning, highlighted the tasks’ high clarity and intuitive design. The high ratings for “Efficiency” and “Dependability” underscored the IVR task’s perceived effectiveness, speed, and reliability. Additionally, high ratings for the qualities of “Stimulation” and “Novelty” pointed to an engaging, exciting, and innovative interactive experience.

Similarly, the SUS data indicated an overwhelmingly positive overall evaluation of the IVR procedure. Participants strongly agreed that the tasks were easy to use and quickly learned, suggesting a low cognitive barrier to entry and rapid user proficiency. High participant confidence and well-integrated functions further emphasized the coherent and user-friendly design of the IVR tasks. Moreover, negative usability items—such as unnecessary complexity and inconsistency—received consistently low ratings. High ratings for the provided instructions also suggest an effective onboarding process.

Overall, the consistently high ratings observed for usability, user experience, and acceptability reflect the successful application of user-centered design principles in the IVR procedure’s development [39]. These positive findings are largely in line with previous studies that have reported high rates of usability and engagement for cognitive tasks utilizing IVR technology in young participants [40,41,42,43].

Specifically, our results align with the literature consistently reporting the preference and high usability of Head-Mounted Displays (HMDs) in the cognitive assessment of young adults. This preference is often attributed to increased motivation [41,42,44], more intuitive action control, and greater enjoyment associated with task fulfillment, e.g., [45]. While these studies focused exclusively on young subjects, making their findings difficult to generalize to the senior population, some evidence suggests that high immersion is well tolerated. For instance, a study by Plechatà et al. [46] demonstrated that evaluated user experiences did not differ between platforms, and seniors reported only minimal and rare side effects. This implies that highly immersive technology holds strong potential for acceptance among aging adults.

Moreover, the positive evaluations observed across all participants, regardless of their self-reported gaming or technology experience, indicate that the IVR procedure is highly accessible and user-friendly. This is a significant achievement, suggesting that the IVR training can be effectively used in diverse populations, including those who may not be familiar with VR technology, such as older adults [39,40,41,42,43,44,45,46], which is a crucial consideration for large-scale application in clinical or community settings.

The findings from the NASA-TLX suggest that while the IVR procedure required a notable degree of mental effort and active engagement from participants, it is generally perceived as manageable, achievable, and not overly frustrating. In particular, results of the “Mental Demand” subscale revealed that participants perceived the IVR tasks as cognitively demanding. However, ratings of the “Own Performance” dimension indicate that, despite the perceived mental demand, a large percentage of participants felt they were performing effectively or very effectively, suggesting that the cognitive resources expended were generally sufficient for successful task completion. The “Effort” subscale corroborated these findings, showing that participants actively engaged in the task and committed significant resources to achieve their objectives. High perceived “Effort” further corroborated the active cognitive engagement. Crucially, “Physical Demand”, as expected, was very low, confirming the predominantly cognitive nature of the IVR tasks. “Temporal Demand” also indicated low time pressure for the majority. Moreover, the “Frustration Level” remained remarkably low, indicating that despite the mental effort, the IVR tasks generally did not induce significant negative emotional responses. These findings suggest that the IVR task effectively balances cognitive challenge with participant support, preventing frustration even under higher mental demands.

Results from the Simulator Sickness Questionnaire (SSQ) completed this workload insight. This outcome is fundamental for the validation of the proposed IVR procedure, as the literature reports that simulator sickness is a condition that can affect individuals when they interact with IVR environments [47]. Our results demonstrated a remarkably low incidence of IVR-induced side effects. The vast percentage of participants reported individual symptoms as “Absent” or “Slight” and no participant asked to terminate their participation in the study. Only a small minority of participants reported negative symptoms rated as moderate or high severity, and only on a few specific items (i.e., “Blurred vision,” “Fullness of Head,” and “Difficulty focusing”). Overall, these findings—suggesting a minimal occurrence and low symptom ratings for cybersickness associated with our IVR procedure—are in line with previous literature reporting no or minimal negative side effects in young adults when performing cognitive tasks in IVR [48,49,50]. The literature has consistently reported that repeated exposure to immersive virtual reality can lead to a reduction in its adverse effects [51,52]. In this regard, it is interesting to note that, despite the majority of our sample having limited or no prior experience with HMDs or other immersive virtual reality devices, the reported side effects associated with the IVR experience were overall very small. This positive outcome can be attributed to the design of the task. Since the literature indicates that motion and visual-vestibular mismatch are major triggers for adverse IVR-induced side effects [53], we deliberately avoided self-motion and required participants to perform all tasks while seated comfortably to mitigate these triggers. However, these results were obtained from a sample of healthy young adults, whereas our target population for cognitive training consists of elderly individuals with SCD [27]. As older adults may have different susceptibilities to cybersickness, further evidence is warranted to confirm the safety and tolerability of this procedure in the intended user population.

### 4.2. Participants’ Performance on IVR Tasks

The results from both the Virtual Face Name Memory Task and the Virtual Object Location Memory Task provide preliminary insights into participants’ cognitive performance on the IVR tasks. In the immediate recognition of the Virtual Face Name Memory Task Part 1, participants achieved high accuracy in the three-alternative forced-choice task for both names (80%) and occupations (95%) associated with virtual faces, demonstrating a strong capacity for associative learning. A reduction in performance was subsequently observed in the delayed phase (Part 2), which followed a 10 min interval. In fact, in the delayed cued-recall test format, accuracy for names dropped to 49% and for occupations to 59%, indicative of typical memory decay and potential retroactive interference from intervening activities [27,39,40]. Nevertheless, memory performance notably improved in the delayed recognition task, with accuracy reaching 76% for names and 88% for occupations. This finding highlights the classic distinction between recall and recognition memory, demonstrating that while the memory traces for these associations may weaken over time, they remain accessible when supported by recognition cues [40,41].

Similarly, performance in the Virtual Object Location Memory Task demonstrated a high capacity for visual and spatial working memory processing. In the change-detection test format, participants exhibited high accuracy in both identifying that a change had occurred (88–92%) and correctly localizing the changed objects (90–95%) across all difficulty levels (Level 1 with 3 objects to Level 3 with 5 objects). High performance was also observed in the subsequent yes/no recognition plus location memory test format, with high accuracy across levels for both object recognition (89–97%) and for objects’ original locations recall (81–95%).

Beyond these high accuracy scores, a critical observation emerged from task progression (Figure 9, Right Panel). In this time-limited task, the percentage of participants who successfully completed each progressive difficulty level decreased sharply as the number of objects increased. Accordingly, while the high mean percentage of correct responses for Level 4 (94%) and Level 5 (97%) was indicative of the performance of almost the entire sample (94% and 83% of participants, respectively), the high accuracy observed in Levels 6 (97%) and 7 (89%) reflected the performance of a progressively smaller and more proficient subset of the sample (60% and 20% of participants, respectively).

### 4.3. Limitations

Several methodological limitations should be acknowledged in interpreting the present findings. The first limitation relates to the sample characteristics: the feasibility and acceptability of the proposed IVR tasks were tested exclusively in a sample of young and healthy subjects. Consequently, we can only speculate that similar results concerning user experience and usability will be observed in our target clinical population (individuals with SCD). Furthermore, the relatively small sample size, although appropriate for a pilot study focused on usability and feasibility as discussed elsewhere [35,43], must be considered a limitation when interpreting the generalizability of our findings. A second limitation is the absence of a control group. Actually, this choice was due to the exploratory nature of this pilot study, which was primarily focused on conducting a preliminary evaluation of the proposed VR cognitive tasks. Accordingly, our aim was to investigate the usability and feasibility of the system, highlighting potential issues that needed to be addressed and improved before administration on the target clinical population.

## 5. Conclusions

In conclusion, this pilot study provides compelling preliminary evidence for the usability and feasibility of two novel IVR memory tasks for cognitive training. The overwhelmingly positive user experience, coupled with low perceived frustration and a minimal incidence of simulator sickness, confirms that our IVR procedure could be a feasible and engaging tool for cognitive intervention. However, these findings should be interpreted in light of the study’s methodological limitations (e.g., reduced sample size, sample composed exclusively of young, healthy subjects, absence of a control group), which restrict the generalizability of the results to the target population (individuals with SCD). These limitations provide the essential basis for future investigations.

By successfully balancing high usability with targeted cognitive demands, these IVR tasks present a robust, user-centric foundation for a planned randomized controlled trial. The results of this study will be instrumental in implementing the 5-week cognitive training program for individuals with Subjective Cognitive Decline [27], ensuring the intervention can be both effective and well received by its target population.

## Data Availability

The data presented in this study are available on request from the corresponding author due to privacy, legal, and ethical reasons.

## Supplementary Materials

The following supporting information can be downloaded at: https://www.mdpi.com/article/10.3390/brainsci15121289/s1, Post-experimental questionnaires; Table S1: Mean, Standard Deviation and Standard error for all dimensions of USEQ questionnaire; Table S2: Mean, Standard Deviation and Standard error for all dimensions of NASA-TLX questionnaire; Table S3: Mean and Variance for all dimensions of UEQ questionnaire; Table S4: Mean, Standard Deviation and Standard error for all items of SUS questionnaire; Table S5: Mean, Standard Deviation and Standard error for all symptoms of SSQ questionnaire; Table S6: Mean, Standard Deviation and Standard error for subscales and total score of SSQ questionnaire.
