# Supplementary material for: User Experience, System Usability, and Feasibility of Two Novel Immersive Virtual Reality Memory Tasks for Cognitive Training: A Pilot Study
Source: Brain Sci. 2025 Nov 29;15(12):1289. doi: 10.3390/brainsci15121289 (PMC12730937; doi:10.3390/brainsci15121289)
Supplement: Supplementary file 1 [file brainsci-15-01289-s001.zip › brainsci-3945754-supplementary.pdf]

Supplemental Materials

Post-experimental questionnaires

USEQ	Mean	Dev st	Err
Experienced enjoyment	4,70	0,53	0,09
Successful use	4,64	0,55	0,10
Ability to control	4,73	0,45	0,08
Clarity of information	4,94	0,24	0,04
Discomfort	2,09	1,18	0,21
Perceived utility	4,27	0,88	0,15

Tab 1 | Mean, Standard Deviation and Standard error for all dimensions of USEQ questionnaire

NASA-TLX	Mean	Dev st	Err
Mental Demand	71,76	19,81	3,45
Physical Demand	16,15	15,65	2,72
Temporal Demand	32,52	24,44	4,26
Own Performance	74,55	18,56	3,23
Effort	60,58	25,75	4,48
Frustration Level	16,12	18,50	3,22

Tab 2 | Mean, Standard Deviation and Standard error for all dimensions of NASA-TLX questionnaire

UEQ Scales (Mean and Variance)		
Attractiveness	1,884	0,72
Perspicuity	2,364	0,59
Efficiency	1,826	0,77
Dependability	1,311	0,64
Stimulation	1,939	0,84
Novelty	2,159	0,62

Tab 3 | Mean and Variance for all dimensions of UEQ questionnaire

SUS	Mean	Dev st	Err
I think that I would like to use this system frequently	4,09	0,88	0,15
I found the system unnecessarily complex.	1,55	0,94	0,16
I thought the system was easy to use.	4,64	0,55	0,10
I think that I would need the support of a technical person to be able to use this system	2,73	1,28	0,22
I found the various functions in this system were well integrated	4,45	0,62	0,11
I thought there was too much inconsistency in this system	1,27	0,67	0,12
I would imagine that most people would learn to use this system very quickly	4,67	0,60	0,10
I found the system very cumbersome to use.	2,00	1,09	0,19
I felt very confident using the system	4,15	0,94	0,16
I needed to learn a lot of things before I could get going with this system	1,25	0,44	0,08
Were the instructions provided sufficient to enable system utilization?	4,76	0,75	0,13
Was the period of VR headset wear during training unduly prolonged?	1,45	0,56	0,10

Tab 4 | Mean, Standard Deviation and Standard error for all items of SUS questionnaire

SSQ	Mean	Dev st	Err
General Discomfort	1,12	0,33	0,06
Fatigue	1,33	0,60	0,10
Headache	1,39	0,61	0,11
Eye Strain	1,61	0,70	0,12
Difficulty Focusing	1,82	0,81	0,14
Salivation Increasing	1,03	0,17	0,03
Sweating	1,15	0,44	0,08
Nausea	1,15	0,36	0,06
Difficulty Concentrating	1,42	0,71	0,12
Fullness of Head	2,09	0,98	0,17
Blurred Vision	1,61	0,83	0,14
Dizziness with Eye Open	1,09	0,29	0,05
Dizziness with Eye Closed	1,15	0,44	0,08
Vertigo	1,06	0,35	0,06
Stomach Awareness	1,03	0,17	0,03
Burping	1,03	0,17	0,03

Tab 5 | Mean, Standard Deviation and Standard error for all symptoms of SSQ questionnaire

SSQ			
**Subscale**	**Mean**	**Dev St**	**Err**
Nausea	8,96	10,64	1,85
Oculomotor	25,04	21,14	3,68
Disorientation	41,34	31,22	5,43
**Total Score**	26,97	20,75	3,61

Tab 6 | Mean, Standard Deviation and Standard error for subscales and total score of SSQ questionnaire
